# Association of Access to Family Planning Services With Medicaid Expansion Among Female Enrollees in Michigan

**DOI:** 10.1001/jamanetworkopen.2018.1627

**Published:** 2018-08-31

**Authors:** Michelle H. Moniz, Matthias A. Kirch, Erica Solway, Susan D. Goold, John Z. Ayanian, Edith C. Kieffer, Sarah J. Clark, Renuka Tipirneni, Jeffrey T. Kullgren, Tammy Chang

**Affiliations:** 1 Department of Obstetrics and Gynecology, University of Michigan, Ann Arbor; 2 Institute for Healthcare Policy and Innovation, University of Michigan, Ann Arbor; 3 School of Public Health, University of Michigan, Ann Arbor; 4 Department of Internal Medicine, University of Michigan, Ann Arbor; 5 Center for Bioethics and Social Sciences in Medicine, University of Michigan, Ann Arbor; 6 School of Social Work, University of Michigan, Ann Arbor; 7 Department of Pediatrics, University of Michigan, Ann Arbor; 8 Veterans Affairs Ann Arbor Center for Clinical Management Research, University of Michigan, Ann Arbor; 9 Department of Family Medicine, University of Michigan, Ann Arbor

## Abstract

**Importance:**

To date, 32 states and the District of Columbia have expanded Medicaid programs under the Patient Protection and Affordable Care Act. It is vital to understand whether expanded health insurance coverage of low-income individuals improves access to family planning services as a first step toward improving reproductive health outcomes.

**Objective:**

To evaluate the association of Medicaid expansion coverage with access to birth control and family planning services among women of reproductive age enrolled in the Michigan expansion plan.

**Design, Setting, and Participants:**

In a survey study, from January 13 through December 15, 2016, telephone surveys of a stratified sample of enrollees in Michigan’s Section 1115 Medicaid Expansion waiver program, the Healthy Michigan Plan (HMP), were conducted. Interviewers completed surveys for 4090 sampled enrollees, of whom 1166 were women aged 19 to 44 years. Surveys were conducted with a computer-assisted telephone interviewing system in English, Arabic, and Spanish. The sample was weighted to 113 565 women. Dates of data analysis were from January 27 through September 18, 2017.

**Main Outcomes and Measures:**

Self-reported change in access to birth control and family planning services through HMP (better, worse, about the same, or don’t know/doesn’t apply), compared with before enrollment.

**Results:**

Among the 1166 survey respondents aged 19 to 44 years (mean [SD] age, 31.0 [0.3] years) and the weighted sample of 113 565, 74.7% (95% CI, 72.2%-76.9%) lived in very-low-income households (<100% federal poverty level), 64.0% (95% CI, 60.5%-67.3%) reported at least 1 chronic medical condition, 23.5% (95% CI, 20.6%-26.6%) reported fair or poor health, and 17.7% (95% CI, 15.7%-19.9%) lived in rural settings. Overall, 35.5% (95% CI, 32.2%-39.0%) reported increased access to family planning services. After adjusting, those most likely to report increased access were women without health insurance coverage in the year preceding HMP enrollment (adjusted odds ratio [aOR], 2.02; 95% CI, 1.41-2.89) compared with women with health insurance for the full 12 months preceding enrollment; younger women (aOR for 19-24 years, 2.80 [95% CI, 1.75-4.50]; aOR for 25-34 years, 2.35 [95% CI, 1.60-3.45]) compared with women aged 35 to 44 years; and women with a recent visit to a primary care clinician (aOR 1.69; 95% CI, 1.03-2.76) compared with women without a primary care visit in the preceding 12 months.

**Conclusions and Relevance:**

One in 3 women of reproductive age reported better ability to access birth control and family planning services through HMP compared with before enrollment. This finding suggests that Medicaid expansion is associated with improved access to family planning services, which may enable low-income women to maintain optimal reproductive health.

## Key Points

**Question:**

Did Medicaid expansion in Michigan improve access to birth control and family planning services?

**Findings:**

In this survey study of 1166 female Medicaid expansion enrollees of reproductive age in Michigan (sample weighted to 113 565 women), 35.5% reported increased access to birth control and family planning services. Those most likely to report increased access were women aged 19 to 24 (39.8%) and 25 to 34 (41.4%) years, women without health insurance coverage in the year preceding Medicaid expansion enrollment (42.6%), and women with a recent visit to a primary care clinician (36.8%).

**Meaning:**

Results suggest that Medicaid expansion is associated with improved access to family planning services, which may enable low-income women to maintain optimal reproductive health.

## Teaser

This survey study evaluates the association of Medicaid expansion coverage with improved access to birth control and family planning services among women of reproductive age recently enrolled in the Healthy Michigan Plan.

## Introduction

Michigan is among the 32 states and District of Columbia that have expanded their Medicaid programs under the Patient Protection and Affordable Care Act (ACA).^1^ Michigan’s Section 1115 Medicaid Expansion waiver program, the Healthy Michigan Plan (HMP), began enrolling patients in April 2014 and now provides health insurance to approximately 650 000 low-income adults, including approximately 200 000 Michigan women of reproductive age.^2,3^ It is critical to understand the effects of Medicaid expansion programs, which the ACA requires to cover birth control without cost-sharing, on access to care.^4^

Contraceptive care is an essential health service for women of reproductive age.^5^ Approximately 45% of US pregnancies are unintended, with an even higher proportion (55.9%) classified as unintended among low-income women (<150% federal poverty level).^6^ Unintended pregnancies generate an estimated $5 billion in direct and indirect health care costs each year in the United States, and 68% of care associated with unintended pregnancies is funded through Medicaid and other public programs.^7,8^ Improving consistent use of effective contraceptive methods is the main approach to reducing unintended pregnancies and their consequences. An estimated 37.9 million women in the United States are currently at risk of unintended pregnancy—that is, they are sexually active, physically able to conceive, and not pregnant or trying to conceive.^9^ Contraceptive need is particularly acute among low-income women, who are more likely than high-income women to report nonuse of contraceptives, to use less effective contraceptive methods, and to experience a contraceptive failure.^10,11,12^

What impedes contraceptive use? Most forms of birth control require a prescription (eg, contraceptive pills, patch, ring) or clinician administration or insertion (eg, contraceptive shot, intrauterine devices, contraceptive implant). The most effective contraceptive methods—including intrauterine devices, implants, and sterilization—are expensive but also cost-effective.^13^ Before Medicaid expansion, many low-income women had significant out-of-pocket costs for contraception, which left some women using less effective contraceptive methods or no method at all. Expanded health insurance coverage of low-income women may remove some critical barriers to contraceptive care, such as out-of-pocket costs for contraceptive methods and visits. However, expanded coverage may not translate into improved access to contraceptive services because of nonfinancial access barriers such as inadequate sources of reproductive health care, discomfort with health care clinicians, logistical barriers (eg, child care, time off work, transportation), distance to a trained reproductive health clinician, and misinformation or poor health literacy.^14,15^ It is critically important to understand whether expanded health insurance coverage of low-income individuals improves access to family planning services as a first step toward reducing unintended pregnancy and improving reproductive health outcomes. Our study aimed to evaluate the association of obtaining Medicaid expansion coverage with access to birth control and family planning services among women enrolled in Michigan.

## Methods

### Study Design

We conducted a telephone survey of HMP enrollees as part of the formal evaluation of the Medicaid Section 1115 demonstration under contract with the Michigan Department of Health and Human Services. This study evaluating a public program followed the American Association for Public Opinion Research (AAPOR) reporting guidelines and was deemed exempt from review by the institutional review boards of the University of Michigan and Michigan Department of Health and Human Services and did not require informed consent.

### Survey Sampling and Administration

From January 13 through December 15, 2016, a stratified sample of HMP enrollees was drawn each month; the sample was allocated proportionally to the overall HMP enrollment by geographic region (Northern Michigan, Central Michigan, Southern Michigan, and Detroit) and income (0%-35%, 36%-99%, and 100%-133% of the federal poverty level). Inclusion criteria were based on demographic characteristics available in the Michigan Department of Health and Human Services data warehouse at the time of sampling and included ages 19 to 64 years; initial HMP enrollment at least 12 months before sampling; enrollment in an HMP managed care plan for at least 9 months (because most HMP enrollees are in managed care, and those who are not are not representative of the typical HMP experience); preferred language of English, Spanish, or Arabic; and a complete Michigan address and telephone number. Sampled HMP enrollees were mailed a letter and brochure that described the project and indicated the project team would call to discuss the survey; enrollees were also given the option to indicate their preferred time of day for the survey via postage-paid postcard, email, or toll-free number. The letter and brochure included general descriptions of the survey project, using language such as:

Healthy Michigan Voices is a phone survey conducted by the University of Michigan. It includes people like you, who are enrolled in the Healthy Michigan Plan or other health plan. The survey asks about doctor and dentist visits, health care costs, and ways in which the Healthy Michigan Plan is working for you.

Project interviewers called sampled HMP enrollees on weekdays from 9 am to 9 pm or at the enrollees’ requested time. Surveys were conducted with a computer-assisted telephone interviewing system in English, Arabic, and Spanish. As an institutional review board–exempt project, written informed consent was not required, but interviewers provided potential participants with information about the survey, ability to stop at any time, and confidentiality of responses, and individuals could then choose whether to participate. Respondents received a $25 gift card incentive. Of the 9227 HMP enrollees who were mailed the initial recruitment materials, 4108 completed the survey (weighted response rate, 53.7% using the American Association for Public Opinion Research’s response rate formula 3).^16^ Eighteen surveys had more than 20% missing data and were excluded from further analysis. Of the remaining 4090 respondents, 1681 were men and 2409 were women. Of the 2409 women, 1166 were aged 19 to 44 years. The analytic sample for the present study was limited to these 1166 female survey respondents aged 19 to 44 years, based on national guidelines for monitoring access to contraceptive care.^17^ The respondents were weighted to a population of 113 565 women. Pregnant women seeking Medicaid coverage are not eligible for HMP, are enrolled in a different Medicaid program, and therefore are not included in our sample.

### Survey Instrument

The survey was part of a larger evaluation of HMP, as required by the Centers for Medicare & Medicaid Services.^18^ To inform survey development, we conducted in-depth semistructured interviews with 67 HMP enrollees from April 1 through August 31, 2015. Interview participants with at least 6 months of HMP enrollment and who had used at least 1 HMP-covered health care service were recruited through community outreach efforts, and purposive sampling methods were used to select interviewees with a diversity of age, race/ethnicity, income, health conditions, geographic region, and urban or rural residence. Guided by findings from these interviews, the survey instrument was developed by the research team.

The survey measured demographics, health status, insurance status, health care access, and use of health care services with established items and scales.^19,20,21,22^ When established measures were not available, new items were developed based on findings from the qualitative interviews. New items underwent cognitive testing and pretesting before being included in the survey instrument. Given the well-documented disparities in contraceptive access across racial/ethnic groups,^23,24,25^ we collected information on race/ethnicity. Respondents were asked “Are you Hispanic or Latino (yes/no)?” “Are you of Arab or Chaldean or Middle Eastern descent (yes/no)?” and “What race or races do you consider yourself to be?” (options defined by respondent). Primary care is an important and unique feature of Michigan’s Medicaid expansion. Enrollees in HMP are encouraged to schedule an appointment with their primary care clinician within 60 days of choosing or being assigned to a health plan. We therefore also collected information about use of primary care services (“Have you seen your primary care provider in the past 12 months?”).

The final instrument also included 1 item addressing access to different categories of health care, including family planning services. Female respondents aged 19 to 44 years were read the following prompt:

Next I’m going to ask about different categories of health care. Tell me if your ability to get that type of care through the Healthy Michigan Plan is better, worse, or about the same, compared to before you had Healthy Michigan Plan. You can also say if you don’t know, or if that type of care doesn’t apply to you. Would you say that your ability to get birth control/family planning services through the Healthy Michigan Plan is better, worse, or about the same, compared to before?

Respondent replies were recorded as better, worse, about the same, or don’t know/doesn’t apply.

### Statistical Analysis

We used descriptive statistics and Pearson χ^2^ analyses to describe self-reported changes in access to reproductive services by demographic and insurance characteristics. We also performed a logistic regression model that estimated a positive change in access to family planning services, including age, race/ethnicity, income, urbanicity, marital status, chronic disease, insurance coverage before HMP, and visit with a primary care clinician in the past 12 months as covariates. A sensitivity analysis was performed excluding those who responded “don’t know/doesn’t apply.” Adjusted odds ratios (aORs) and their 95% CIs were calculated. Item nonresponses were coded as missing. All analyses were weighted using the svy: command in Stata software (version 14.2; StataCorp). Survey selection weight, adjustments for nonworking numbers, ineligible cases, unknown eligibility, and nonresponse, as well as poststratification weights and sampling strata were applied to adjust for sample design and nonresponse. All statistics applied these weights, and the resulting statistics reflect the overall HMP population. Two-sided *P* < .05 was considered statistically significant.

## Results

Our sample of 1166 female respondents (weighted population, 113 565 women) aged 19 to 44 years (mean [SD] age, 31.0 [0.3] years) broadly reflected the racial/ethnic composition of low-income nonelderly adults in Michigan (Table 1). Most women (74.7%; 95% CI, 72.2%-76.9%) lived in very-low-income households (<100% of the federal poverty level). At least 1 chronic medical condition was reported by 64.0% (95% CI, 60.5%-67.3%), and 23.5% (95% CI, 20.6%-26.6%) reported fair or poor health. Nearly 1 in 5 (17.7%; 95% CI, 15.7%-19.9%) lived in rural settings.

**Table 1.** Characteristics of Female Respondents Aged 19 to 44 Years

Variable^a^	Unweighted No. of Respondents (n = 1166)^b^	Weighted Proportion, % (95% CI)
Age, y		
19-24	245	23.8 (20.8-27.1)
25-34	509	44.4 (40.9-47.9)
35-44	412	31.8 (28.6-35.2)
Race/ethnicity		
Non-Hispanic white	748	59.9 (56.4-63.4)
Non-Hispanic black	249	24.6 (21.5-27.9)
Hispanic	24	2.2 (1.4-3.3)
Other	139	13.4 (11.1-16.0)
FPL category, %		
0-35	312	40.2 (36.8-43.6)
36-99	488	34.5 (31.8-37.3)
≥100	366	25.3 (23.1-27.8)
Married or partnered		
Yes	336	23.7 (21.2-26.4)
No	828	76.3 (73.6-78.8)
Urban county		
Rural	299	17.7 (15.7-19.9)
Urban	867	82.3 (80.1-84.3)
Health status		
Excellent, very good, or good health	904	76.5 (73.4-79.4)
Fair or poor health	262	23.5 (20.6-26.6)
Any chronic health condition		
Yes	755	64.0 (60.5-67.3)
No	411	36.0 (32.7-39.5)
Insurance the year before HMP enrollment		
All 12 mo	434	37.7 (34.2-41.2)
Some of year	129	12.4 (10.1-15.1)
Uninsured all 12 mo	568	50.0 (46.4-53.6)
Visit with primary care clinician in past 12 mo		
Yes	945	85.9 (83.1-88.2)
No	146	14.1 (11.8-16.9)

Abbreviations: FPL, federal poverty level; HMP, Healthy Michigan Plan.

^a^ Variables derived from the data warehouse at time of sampling include age, FPL categories, and urban county. All other variables derived from survey responses.

^b^ Owing to missing data, not all categories total 1166 respondents.

Overall, 35.5% (95% CI, 32.2%-39.0%) of respondents reported better ability to access birth control and family planning services through HMP compared with before they enrolled in HMP, whereas 24.8% (95% CI, 21.8%-28.0%) reported about the same ability, and 1.4% (95% CI, 0.8%-2.5%) reported worse ability. An additional 38.3% (95% CI, 34.9%-41.8%) reported that they did not know whether HMP affected their ability to get birth control and family planning or that birth control and family planning access did not apply to them. The proportion reporting improved access to family planning services was lower than the proportion of women aged 19 to 44 years reporting improved access to primary care, specialist care, dental care, prescription medications, and help preventing health problems, but higher than the proportion reporting improved access to mental health, cancer screening, and substance use treatment (Figure).

**Figure.**
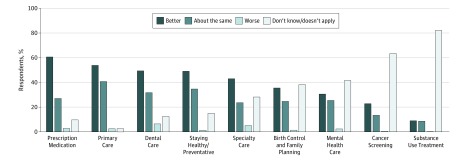
Self-reported Change in Access to Health Care Services After Healthy Michigan Plan Enrollment Proportions are weighted to a population of 113 565 women.

Significantly higher proportions of younger women aged 19 to 24 years (39.8%; 95% CI, 32.7%-47.4%) and aged 25 to 34 years (41.4%; 95% CI, 36.3%-46.8%) reported improved ability to obtain birth control and family planning services after acquiring HMP coverage compared with women aged 35 to 44 years (24.1%; 95% CI, 19.4%-29.6%; *P* < .001) (eTable in the Supplement). Improved access to birth control and family planning services was also more commonly reported by women without health insurance in the year preceding HMP coverage (42.6%; 95% CI, 37.7%-47.6%) compared with those who were insured all 12 months (27.5%; 95% CI, 22.3%-33.2%; *P* = .001). A difference in access to family planning services was observed for women who had seen a primary care clinician in the last 12 months, compared with those without a primary care visit, but the difference did not achieve significance (36.8% [95% CI, 33.1%-40.8%] vs 27.6% [95% CI, 19.9%-36.7%]; *P* = .06).

In multivariable logistic regression analysis, better access to birth control and family planning services was significantly associated with age, having no health insurance coverage before HMP enrollment, and a recent visit with a primary care clinician (Table 2). Compared with enrollees aged 35 to 44 years, younger women had significantly higher odds of reporting better access to birth control and family planning services (aOR for 19-24 years, 2.80 [95% CI, 1.75-4.50]; aOR for 25-34 years, 2.35 [1.60-3.45]). Compared with women with health insurance for the full year before enrolling in HMP, women without any insurance in the preceding 12 months had twice the odds of reporting that HMP improved their access to birth control and family planning services (aOR, 2.02; 95% CI, 1.41-2.89). Enrollees who had visited a primary care clinician in the preceding 12 months also had significantly higher odds of reporting better access to birth control and family planning after HMP enrollment compared with those who had not (aOR, 1.69; 95% CI, 1.03-2.76). We did not observe differences in self-reported access across racial/ethnic groups, income categories, partnership status, urban or rural setting, self-reported health status, or presence of medical comorbidity. In the sensitivity analysis, after dropping responses of “don’t know/doesn’t apply,” women without health insurance in the past 12 months were significantly more likely to report improved access to family planning services (aOR, 3.11; 95% CI, 1.99-4.86).

**Table 2.** Unadjusted Proportions and Adjusted Odds of Self-reported Improvement in Access to Family Planning Services

Respondent Characteristic (No. of Respondents)	Unadjusted Weighted % (95% CI)	aOR (95% CI)^a^
Age, y^b^		
19-24 (n = 244)	39.8 (32.7-47.4)	2.80 (1.75-4.50)
25-34 (n = 509)	41.4 (36.3-46.8)	2.35 (1.60-3.45)
35-44 (n = 411)	24.1 (19.4-29.6)	1 [Reference]
All (n = 1164)	35.5 (32.2-39.0)	NA
Race/ethnicity		
Non-Hispanic white (n = 746)	34.1 (30.0-38.4)	1 [Reference]
Non-Hispanic black (n = 249)	35.3 (28.2-43.1)	1.11 (0.71-1.73)
Hispanic (n = 24)	46.3 (26.5-67.3)	1.39 (0.55-3.49)
Other (n = 139)	42.5 (33.2-52.4)	1.38 (0.84-2.28)
All (n = 1158)	35.8 (32.4-39.3)	NA
FPL category, %		
0-35 (n = 311)	34.8 (28.7-41.4)	1 [Reference]
36-99 (n = 488)	37 (32.1-42.3)	1.21 (0.80-1.80)
≥100 (n = 365)	34.7 (29.4-40.4)	1.13 (0.74-1.72)
All (n = 1164)	35.5 (32.2-39.0)	NA
Married or partnered		
Yes (n = 336)	34.2 (28.6-40.2)	1 [Reference]
No (n = 826)	36.1 (32.1-40.3)	1.00 (0.70-1.42)
All (n = 1162)	35.6 (32.3-39.1)	NA
County		
Rural (n = 299)	35.6 (29.7-41.9)	1 [Reference]
Urban (n = 865)	35.5 (31.7-39.6)	1.03 (0.71-1.49)
All (n = 1164)	35.5 (32.2-39.0)	NA
Health status		
Excellent, very good, or good health (n = 902)	35.3 (31.6-39.3)	1 [Reference]
Fair or poor health (n = 262)	36.2 (29.2-43.9)	1.09 (0.72-1.65)
All (n = 1164)	35.5 (32.2-39.0)	NA
Any chronic health condition		
Yes (n = 754)	35.5 (31.3-40.0)	1 [Reference]
No (n = 410)	35.6 (30.3-41.2)	1.19 (0.82-1.72)
All (n = 1164)	35.5 (32.2-39.0)	NA
Insurance the year before HMP enrollment^b^		
All 12 mo (n = 434)	27.5 (22.3-33.2)	1 [Reference]
Some of year (n = 127)	33.8 (24.4-44.7)	1.29 (0.74-2.25)
Uninsured all 12 mo (n = 568)	42.6 (37.7-47.6)	2.02 (1.41-2.89)
All (n = 1129)	35.8 (32.4-39.3)	NA
Visited primary care clinician in past 12 mo^c^		
Yes (n = 943)	36.8 (33.1-40.8)	1 [Reference]
No (n = 146)	27.6 (19.9-36.7)	1.69 (1.03-2.76)
All (n = 1089)	35.5 (32.1-39.1)	NA

Abbreviations: aOR, adjusted odds ratio; FPL, federal poverty level; HMP, Healthy Michigan Plan; NA, not applicable.

^a^ Indicates odds of reporting better access compared with odds of reporting about the same, worse, or don’t know/doesn’t apply.

^b^ *P* ≤ .001, Pearson χ^2^ test.

^c^ *P* = .06, Pearson χ^2^ test.

## Discussion

In this study examining health care access after Medicaid expansion in Michigan, more than 1 in 3 women of reproductive age reported better ability to access birth control and family planning services through HMP compared with before enrollment. Younger women, those without insurance coverage in the year preceding HMP enrollment, and those with a recent visit to a primary care clinician were significantly more likely to report increased access to family planning services.

Although improved contraceptive access is a critical first step in improving contraceptive use and reducing unintended pregnancy rates, prior studies of the effects of expanded coverage on access to contraceptive services and reproductive health outcomes^26,27,28,29,30,31,32^ have had mixed results. Multiple studies^26,27,28,29^ suggest that Medicaid family planning waivers and state plan amendments to expand family planning coverage to women not otherwise eligible for Medicaid have been associated with increased contraceptive use and significant reductions in unintended pregnancy rates. However, early studies evaluating contraceptive use patterns among commercially and publicly insured women after implementation of the ACA’s contraceptive coverage mandate^30,31,32^ have documented no or only minimal change in contraceptive use patterns. Conflicting findings may be due to variation in women’s baseline contraceptive need at the time of expanded coverage. Our study of Medicaid expansion in Michigan suggests that expansion improves family planning access among low-income women, with the greatest association among young women and those without insurance coverage in the 12 months preceding HMP enrollment, populations that may have had a high unmet need for family planning care. Our findings are consistent with those of a recent literature review of 153 studies of Medicaid expansion^33^ that documents predominantly positive effects of Medicaid expansion on measures of access to health care.

Fewer respondents reported improved access to birth control and family planning compared with some other health care services. This improvement was true even when comparing birth control with other prescription drugs. This finding has several possible explanations. Many low-income women may have already had access to free or low-cost birth control through publicly funded clinics. In 2016, for example, more than 4.0 million individuals were served at approximately 4000 sites funded by the Title X National Family Planning Program, the federal program that supports delivery of family planning care to low-income families.^34^ Some women may not have had a need for birth control or family planning care (eg, they had already received a sterilizing surgery or a long-acting contraceptive device, or they desired pregnancy). Another explanation is that some newly insured individuals may not have known they were eligible for no-cost family planning services.

Young HMP enrollees were significantly more likely to report improved access to family planning services. Young women are at disproportionately high risk of unintended and short-interval pregnancies and associated adverse outcomes, including preterm birth, compared with older women.^6^ Young women face unique barriers to contraceptive access, including concerns about cost and confidentiality.^35^ Provision of no-cost contraception has been shown to decrease unintended pregnancy rates among young women.^36,37,38^ Our findings suggest that Medicaid expansion may help improve family planning access for this vulnerable population with often unmet family planning needs.

Our findings also suggest that primary care clinicians may play an important role in translating insurance coverage into meaningful access to family planning care. Enrollees in HMP with a recent visit to a primary care clinician were significantly more likely to report better birth control and family planning access after HMP enrollment than their peers who had not recently seen a primary care clinician. One explanation is that women who have seen a primary care clinician may have more interest in family planning services. Alternatively, primary care clinicians may inquire about the need for contraception and prescribe birth control or refer new Medicaid enrollees to clinicians with family planning expertise. New enrollees in HMP are encouraged to have an early visit with a primary care clinician, which may be an important strategy for improving access to reproductive health services.^39^ States that have expanded Medicaid can also support clinicians in delivering high-quality family planning care by eliminating multivisit protocols, encouraging contraception access across health care settings, and disseminating the evidence-based guidelines from the Centers for Disease Control and Prevention regarding contraceptive use in women with chronic medical conditions.^40,41,42,43,44,45,46^

We did not observe differences in reported improvement in access to family planning services across racial/ethnic, income, or urban and rural groups. This suggests that the benefits of Medicaid expansion for family planning access are being shared equitably by diverse enrollees.

### Limitations

Our study has several potential limitations. First, we measured only self-reported access to services. Objective measures of appointment availability, service affordability, and clinician acceptability are important topics for future studies of reproductive health access after Medicaid expansion. Second, our survey included adult women of reproductive age. By not specifically excluding women not at risk of unintended pregnancy, we provide conservative estimates of the associations of coverage expansion with access to birth control and family planning services, likely underestimating the association among women at higher risk of unintended pregnancy. Third, the present study focuses on self-reported access to birth control and family planning services. Additional work is needed to understand whether improved access translates into enhanced contraceptive use and improved reproductive health outcomes. Fourth, because we examined Medicaid expansion in a single large state, findings may not be generalizable to all states. State-level evaluations, however, are the most robust method of evaluating Medicaid expansions, given unique implementation processes across states. Michigan’s early experiences may provide useful insights as Medicaid expansion continues to be debated and implemented in other states.

## Conclusions

Our results suggest that Medicaid expansion is associated with improved access to family planning services, which may enable low-income women to maintain optimal reproductive health. States that have adopted or are considering adopting Medicaid expansion can use robust communication efforts to ensure optimal use of family planning services by interested enrollees. Facilitating access to family planning services may improve health outcomes and reduce health care expenditures, because each dollar spent on contraception has been estimated to save the health care system $6.^47,48,49,50^ Our findings provide supporting evidence that Medicaid expansion appears to effectively enhance access to family planning care in Michigan. Further research is required to assess how this increased access affects reproductive health among Medicaid expansion enrollees.

## Notes

Supplement. **eTable.** Healthy Michigan Plan Beneficiary Characteristics and Access to Birth Control and Family Planning Services
