# Supplementary material for: Association of Access to Family Planning Services With Medicaid Expansion Among Female Enrollees in Michigan
Source: JAMA Netw Open. 2018 Aug 31;1(4):e181627. doi: 10.1001/jamanetworkopen.2018.1627 (PMC6324283; doi:10.1001/jamanetworkopen.2018.1627)
Supplement: Supplement. — eTable. Healthy Michigan Plan Beneficiary Characteristics and Access to Birth Control and Family Planning Services [file jamanetwopen-1-e181627-s001.pdf]

Supplementary Online Content

Moniz MH, Kirch MA, Solway E, et al. Association of access to family planning services with Medicaid expansion among female enrollees in Michigan. *JAMA Netw Open.* 2018;1(4):e181627. doi:10.1001/jamanetworkopen.2018.1627

eTable. Healthy Michigan Plan Beneficiary Characteristics and Access to Birth Control and Family Planning Services

This supplementary material has been provided by the authors to give readers additional information about their work.

© 2018 Moniz MH et al. *JAMA Network Open*.

eTable. Healthy Michigan Plan Beneficiary Characteristics and Access to Birth Control and Family Planning Services

	Better		Worse		About the same		Don't know/ Doesn't apply		*P* Value[a]
	Row %	95% CI	Row %	95% CI	Row %	95% CI	Row %	95% CI	
Age									
19-24 (n=244)	39.8	[32.7,47.4]	1.1	[0.3,3.7]	26.7	[20.3,34.2]	32.4	[25.4,40.3]	<.001
25-34 (n=509)	41.4	[36.3,46.8]	2.3	[1.1,4.6]	27.1	[22.9,31.7]	29.2	[24.5,34.4]	
35-44 (n=411)	24.1	[19.4,29.6]	0.3	[0.0,2.4]	20.2	[15.4,26.0]	55.3	[49.1,61.3]	
Total (n=1,164)	35.5	[32.2,39.0]	1.4	[0.8,2.5]	24.8	[21.9,28.0]	38.3	[34.9,41.8]	
Race/ethnicity									
White, non-Hispanic (n=746)	34.1	[30.0,38.4]	1.9	[1.0,3.7]	22.9	[19.4,26.8]	41.1	[36.7,45.6]	.291
Black, non-Hispanic (n=249)	35.3	[28.2,43.1]	0.5	[0.1,3.1]	29.7	[23.2,37.0]	34.6	[27.7,42.2]	
Hispanic (n=24)	46.3	[26.5,67.3]	0		25.2	[11.1,47.8]	28.5	[12.8,51.9]	
Other (n=139)	42.5	[33.2,52.4]	0.9	[0.1,6.3]	25.3	[17.3,35.4]	31.3	[22.8,41.1]	
Total (n=1,158)	35.8	[32.4,39.3]	1.4	[0.8,2.5]	24.9	[22.0,28.2]	37.9	[34.5,41.5]	
FPL categories									
0-35% (n=311)	34.8	[28.7,41.4]	1.9	[0.8,4.7]	21.4	[16.1,27.7]	41.9	[35.3,48.8]	.272
36-99% (n=488)	37	[32.1,42.3]	0.5	[0.2,1.8]	26.3	[22.1,30.9]	36.2	[31.5,41.2]	
>=100% (n=365)	34.7	[29.4,40.4]	1.7	[0.7,4.1]	28.2	[23.3,33.6]	35.5	[30.2,41.1]	
Total (n=1,164)	35.5	[32.2,39.0]	1.4	[0.8,2.5]	24.8	[21.9,28.0]	38.3	[34.9,41.8]	
Married or partnered									
Yes (n=336)	34.2	[28.6,40.2]	1.1	[0.4,2.9]	25.3	[20.4,31.0]	39.5	[33.9,45.4]	.893
No (n=826)	36.1	[32.1,40.3]	1.5	[0.7,3.0]	24.7	[21.2,28.6]	37.7	[33.6,42.0]	
Total (n=1,162)	35.6	[32.3,39.1]	1.4	[0.8,2.5]	24.8	[21.9,28.0]	38.2	[34.7,41.7]	
Urban County									
Rural (n=299)	35.6	[29.7,41.9]	1.6	[0.5,4.5]	28.6	[23.1,34.7]	34.3	[28.6,40.6]	.494
Urban (n=865)	35.5	[31.7,39.6]	1.3	[0.7,2.7]	24	[20.6,27.7]	39.2	[35.2,43.3]	
Total (n=1,164)	35.5	[32.2,39.0]	1.4	[0.8,2.5]	24.8	[21.9,28.0]	38.3	[34.9,41.8]	
Health status									
Excellent, Very Good, or Good Health (n=902)	35.3	[31.6,39.3]	1	[0.5,1.9]	26.4	[23.0,30.2]	37.3	[33.4,41.4]	.115
Fair or poor health (n=262)	36.2	[29.2,43.9]	2.7	[0.9,7.3]	19.5	[14.4,25.9]	41.6	[34.6,48.9]	
Total (n=1,164)	35.5	[32.2,39.0]	1.4	[0.8,2.5]	24.8	[21.9,28.0]	38.3	[34.9,41.8]	
Any chronic health condition present									
Yes (n=754)	35.5	[31.3,40.0]	1.7	[0.8,3.3]	22.3	[18.8,26.2]	40.5	[36.2,45.0]	.094
No (n=410)	35.6	[30.3,41.2]	0.9	[0.3,2.7]	29.3	[24.2,34.9]	34.3	[28.9,40.1]	
Total (n=1,164)	35.5	[32.2,39.0]	1.4	[0.8,2.5]	24.8	[21.9,28.0]	38.3	[34.9,41.8]	
Insurance prior to HMP									
Insured all 12 months (n=434)	27.5	[22.3,33.2]	2.5	[1.1,5.5]	35.3	[30.2,40.9]	34.7	[29.4,40.3]	<.001
Insured some of year (n=127)	33.8	[24.4,44.7]	1	[0.1,6.5]	21.9	[14.5,31.8]	43.3	[33.0,54.2]	
Uninsured all 12 months (n=568)	42.6	[37.7,47.6]	0.5	[0.2,1.3]	18	[14.1,22.6]	38.9	[34.0,44.1]	

© 2018 Moniz MH et al. *JAMA Network Open*.

Total (n=1,129)	35.8	[32.4,39.3]	1.3	[0.7,2.5]	25	[22.0,28.3]	37.8	[34.4,41.4]	
Seen PCP past 12 months									
Yes (n=943)	36.8	[33.1,40.8]	1.2	[0.6,2.3]	24.8	[21.5,28.4]	37.1	[33.3,41.1]	.178
No (n=146)	27.6	[19.9,36.7]	1.2	[0.3,5.2]	24.3	[17.4,33.0]	46.9	[37.5,56.6]	
Total (n=1,089)	35.5	[32.1,39.1]	1.2	[0.7,2.1]	24.8	[21.7,28.1]	38.5	[35.0,42.2]	

[a]Pearson's Chi-square analyses
CI: confidence interval; FPL: federal poverty level; HMP: Healthy Michigan Plan; PCP: primary care provider

© 2018 Moniz MH et al. *JAMA Network Open*.
